# HPV Vaccine Hesitancy Among Medical Students in China: A Multicenter Survey

**DOI:** 10.3389/fpubh.2022.774767

**Published:** 2022-02-21

**Authors:** Liangru Zhou, Jian Wang, Pengxin Cheng, Yue Li, Guoxiang Liu, Xin Zhang

**Affiliations:** School of Health Management, Harbin Medical University, Harbin, China

**Keywords:** HPV vaccine, vaccination information, hesitancy, 3Cs model, medical students

## Abstract

Human papillomavirus (HPV) is the most common genital tract virus infection and can cause genital warts and cervical cancer. This multicenter study examined HPV information sources, vaccine hesitancy, and the association between the two variables. An online survey of HPV information sources and vaccine hesitancy was conducted among Chinese medical students. The World Health Organization (WHO) Vaccine Hesitancy 3Cs model was used to evaluate reasons for respondents' vaccine hesitancy. A probit model was used to investigate the association between vaccine information sources and vaccine hesitancy. The reported rate of vaccine hesitancy was 62.36%. Convenience was the primary factor for vaccine hesitancy in medical students, and 19% used a single source to obtain vaccine information. A multivariate analysis revealed that master degree and above were 33% less likely to be hesitant about the HPV vaccine than first grade students. Respondents receiving HPV information through doctor were 8% less likely to report vaccine hesitancy than those receiving information from other information channels. HPV vaccine hesitancy requires more attention. Future studies could examine whether increasing vaccination locations and dissemination of information about the safety and effectiveness of HPV vaccines as well as using Internet media would help reduce medical students' vaccine hesitancy and expand HPV vaccine coverage.

## Introduction

Human papillomavirus (HPV) is the most common genital tract infection virus. Most sexually active women and men will be infected with the virus at some point in their lives, some repeatedly (1). HPV plays a role in the development of skin diseases such as head and neck cancer, cervical cancer, male genital cancer, and condyloma acuminatum. The HPV subtypes are classified into high-risk (HPVl6, 18, 31, 33, 35, 45, etc.) and low-risk types (HPV6, 11, 30, 39, 42, 43, etc.) (2, 3). Almost all cases of cervical cancer (99%) are associated with high-risk HPV infection (4).

According to the GLOBOCAN 2020 reported that there were 604,000 new cases of cervical cancer and 342,000 deaths worldwide in 2020; 110 thousand new cases of cervical cancer and 59,000 deaths were reported in China, making it the second-largest burden of cervical cancer in the world (5). The prevalence of HPV-related head and neck cancer, especially throat cancer, is increasing rapidly. It is expected that the number of patients with HPV-positive throat cancer will exceed that of patients with cervical cancer in the next 15–20 years (6).

Prophylactic HPV vaccine is an effective way to prevent HPV-related diseases, including cervical cancer (7).The incidence and costs of cervical cancer already significantly decreasing in Italy (8). And vaccination does not require any major lifestyle changes (9).

HPV vaccination has been implemented on a large scale and achieved remarkable results since the vaccine's production and marketing in 2006 (10). Studies in several countries and regions have confirmed that HPV vaccination is cost-effective (11–14).

However, the emergence of vaccine hesitancy has dampened public enthusiasm for vaccination. In 2019, the WHO listed vaccine hesitancy as one of the top 10 threats to global health (15). Vaccine hesitancy refers to either reluctance to accept or rejection of vaccination when it is available (16). A decline in the HPV vaccination rate due to HPV vaccine hesitancy has been reported in many countries and regions. For example, in 2013, after the media reported that vaccination with untested vaccines would cause adverse events, HPV vaccine hesitancy spread widely in Japan, resulting in a significant decline in the Japanese vaccination rate (17). A study of Brazilian medical students suggested that vaccine hesitancy is the fundamental determinant of the low HPV vaccination rate (18). Although studies have been conducted on HPV infection awareness and knowledge in China (19, 20), evidence concerning Chinese HPV vaccine hesitancy is lacking. According to the WHO, confidence is one of the three major determinants of vaccine hesitancy (21). Undoubtedly, unproven negative news has undermined public trust in HPV vaccines. However, for the public, it is challenging to determine authentic and valid facts in the vast pool of available information. Transparent and accurate information is essential for rebuilding vaccine confidence (22). In addition, it is necessary to understand the public's sources of information and improve the efficiency of information transmission.

The HPV vaccine entered the Chinese market as a self-funded vaccine in 2016, with domestic approval of bivalent HPV vaccines for women aged 9–45; quadrivalent HPV vaccines for women aged 20–45; and 9-valent HPV vaccines for women aged 16–26 (23). In November 2020, mainland China reduced the minimum age for quadrivalent vaccines to 9 years. China's HPV vaccine has been on the market for only a few years compared with other countries, and some people adopt the “wait-and-see” approach regarding vaccines, resulting in low vaccination rates. Many medical students are also target populations for the HPV vaccine. In addition, as the future generations of medical personnel, they bear the responsibility of promoting the health of the entire population and play an important role in the promotion and popularization of HPV vaccination. To investigate the factors related to HPV vaccine hesitancy among medical students and estimate the association between the source of information and vaccine hesitancy, the author developed an information source and hesitancy questionnaire and conducted a questionnaire survey with Chinese medical students.

## Materials and Methods

### Study Design and Implementation

At the beginning of November 2020, a preliminary survey was conducted at the author's medical university. The purpose was to improve the preset items and test the practicality of the survey, including whether the question expressions were easy to understand, and to improve the questionnaire through information from pre-survey feedback. The formal survey was conducted from mid-January to the end of March, and a cross-sectional multicenter survey on HPV information sources and hesitancy was conducted for medical students at the school. The investigation was conducted anonymously. Participants were recruited at Harbin Medical University, Hebei Medical University and Chengdu University of Traditional Chinese Medicine. Participants must be students of Chinese nationality. At the beginning of the survey, the participants were asked whether they would voluntarily participate in the research, and indicated that participants are free to withdraw from the research. The questionnaire takes 3–5 min to fill out. Each respondent received 2RMB in cash as remuneration, which was paid through online payment. The study was open to people of any sex. To maintain survey quality, we implemented the following controls: (1) All questions were set as compulsory to avoid missing responses; (2) When a respondent chose the same option for all questions, they were prompted to repeat their answers, and the survey would not proceed until they had done so.

### Data Collection and Questionnaire Measures

A total of 850 medical students were invited. A questionnaire for medical students was compiled, and it included four parts. The first part of the questionnaire reported the purpose and objectives of the research, asked the participants whether they would voluntarily participate in the study, and indicated that they were free to withdraw from the research. The second part of the questionnaire surveyed participants' demographic information, sex, age, grade, type of family residence, partner status, health behavior, whether they had internship experience in a medical institution, and monthly consumption, which was food and shopping expenditures, excluding large expenditures like expensive electronic products.

The third part investigated participants' vaccine hesitancy and their reasons. Vaccine hesitancy refers to delay in acceptance or refusal of vaccines despite availability of vaccine services. Hesitancy was scored as the percentage of participants endorsing each item. The WHO Vaccine Hesitancy 3Cs model classifies the reasons for vaccine hesitancy as complacency, convenience, and confidence. Complacency refers to the underestimation of the harm the disease can cause and doubts about the necessity of vaccine use. Confidence refers to the trust in the safety, effectiveness, health system, and administrators of vaccines. Convenience refers to vaccine supply capacity, vaccine price acceptance, and availability of vaccination services. Following this classification, and in accordance with China's social and cultural environment, 13 possible reasons were included for participants to choose from, and open items were included for participants to add additional reasons.

The fourth part investigated HPV and vaccine information sources. Sources include online media, traditional media represented by newspaper, television and radio, offline publicity such as school lectures and subway advertisements and person-to-person communication among doctors, family, and friends. The number of channels was collected from the results of the information sources and counted according to the reported information sources. The range of the number of channels was 0–8.

### Statistical Analyses

Statistical analysis was performed using Stata 15.0. Categorical variables were analyzed using chi-squared tests, and continuous variables were analyzed using one-way analysis of variance. A probit model was used to identify whether the information source and number of information sources were the key factors for hesitancy, and the marginal utility of the regression coefficients of the probit model was calculated. In the analysis, we controlled for demographic characteristics of the respondents, including age, sex, place of residence (rural or urban), monthly living expenses, and weekly exercise duration. A *P* < 0.05 was considered statistically significant throughout the study.

### Ethics Approval and Consent to Participate

The study was approved by the Medical Ethics Committee of Harbin Medical University (HMUIRB20210006). Respondents were informed that they were free to withdraw from the study and that all data would be strictly confidential and used only for scientific analysis.

## Results

### Participants' Characteristics

A total of 728 respondents responded to the questionnaire. Table 1 shows participants' baseline characteristics. Among the participants, 62% were women, and 38% were men. All were between 16 and 45 years old. A total of 52% of the respondents are masters and above. A total of 78% of respondents lived in urban areas, and 86% did not have a partner. Overall, 85% of them had hospital internship experience, 41% had a monthly consumption of < $300, and 38% exercised <1 h per week.

**Table 1:** Participants' characteristics and vaccine hesitancy.

**Characteristics**	**Sample**	**Hesitancy**
	***N* (%)**	***N* (%)**	***P*-value**
**Total**	728 (–)	454 (–)	–
**Sex**			0.00
Female	450 (62)	258 (57)	
Male	278 (38)	196 (43)	
**Age**			0.07
16–26	538 (74)	325 (72)	
27–45	190 (26)	129 (28)	
**Grade**			0.00
First year of university	35 (5)	31 (7)	
The second year of university	116 (16)	91 (20)	
The third year of university	104 (14)	84 (19)	
Fourth grade of university	65 (9)	37 (8)	
Fifth grade of university	27 (4)	15 (3)	
Master degree and above	381 (52)	196 (43)	
**Type of residence**			0.80
Urban	565 (78)	351 (77)	
Rural	163 (22)	103 (23)	
**Partner**			0.00
No partner	629 (86)	376 (83)	
Have a partner	99 (14)	78 (17)	
**Hospital Internship**			0.42
Yes	621 (85)	391 (86)	
No	107 (15)	63 (14)	
**Monthly consumption (monthly)**			0.06
≤ $300	427 (41)	254 (56)	
>$300	301 (59)	200 (44)	
**Hours of exercise (weekly)**			0.01
≤ 1 h	277 (38)	159 (35)	
1 ≤ 3 h	341 (47)	232 (51)	
>3 h	110 (15)	63 (14)	

### Vaccine Hesitancy and Reasons for Vaccine Hesitancy

Among the respondents, 62.36% (454/728) reported having vaccine hesitancy. Participants of different sexes (*P* = 0.00), different grades (*P* = 0.00), with or without partners (*P* = 0.00), and doing different amounts of exercise (*P* = 0.01) reported different levels of vaccine hesitancy (Table 1).

The reasons for respondents' vaccine hesitancy are shown in Table 2. Overall, 42% of respondents believed that HPV vaccination was unnecessary. Among the convenience items, 63% of participants did not know where to obtain reliable information. Regarding the confidence items, most respondents worried about vaccine safety or side effects (62%), 51% reported negative reactions of others, and 51% reported that they had heard or read negative news.

**Table 2:** Reasons for respondents' vaccine hesitancy.

**Reasons**	**Yes, *N* (%)**
**Complacency**	
No vaccination was considered necessary	190 (42)
**Convenience**	
Don't know where to get vaccinated	264 (58)
The vaccination site is far away and requires a long journey	270 (60)
Repeated vaccination doses, feeling troublesome	274 (60)
Don't know where to get good/reliable information	287 (63)
**Confidence**	
Hear or read negative news	230 (51)
Fear of needles	187 (41)
Someone told me they had a bad reaction	233 (51)
Had a bad experience with previous vaccinations due to health clinics/vaccinators	185 (41)
Worried about vaccine safety/side effects	280 (62)
I've been told that vaccines aren't safe	188 (41)
Vaccination had bad physiological and psychological reactions before	190 (42)
Health care workers' attitudes toward the HPV vaccine	192 (42)

### HPV Information Sources

**Figure 1:**
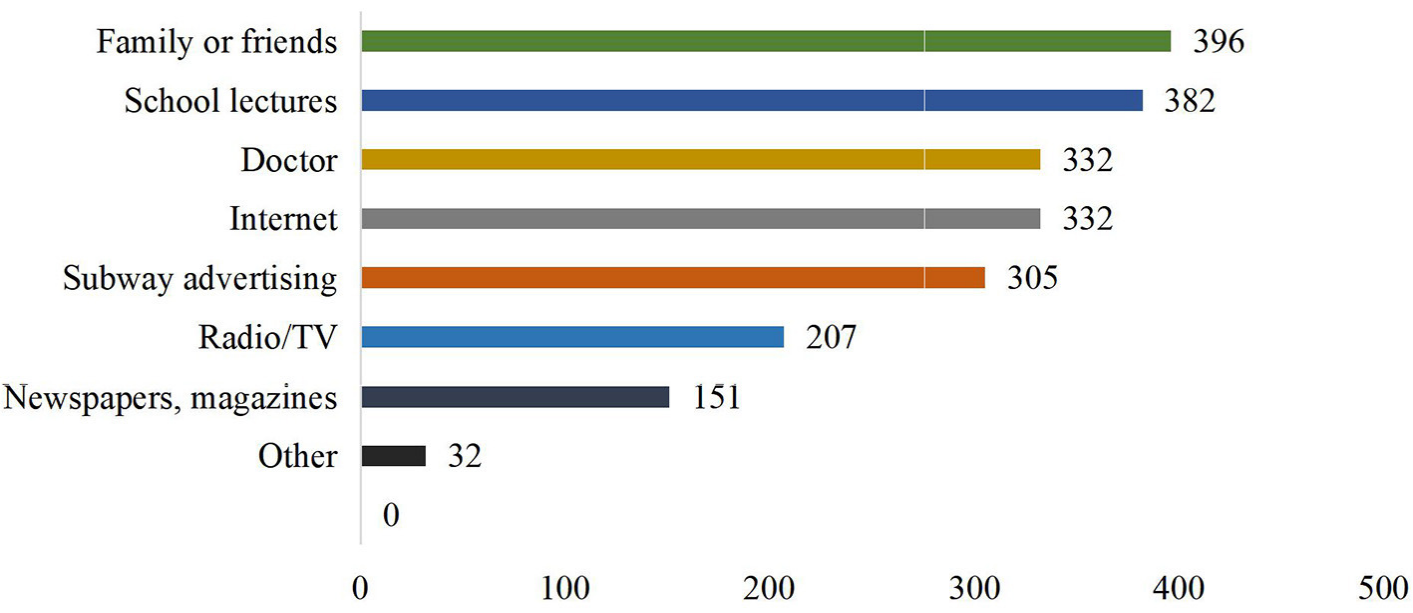
Distribution of respondents' information sources (sorted).

**Figure 2:**
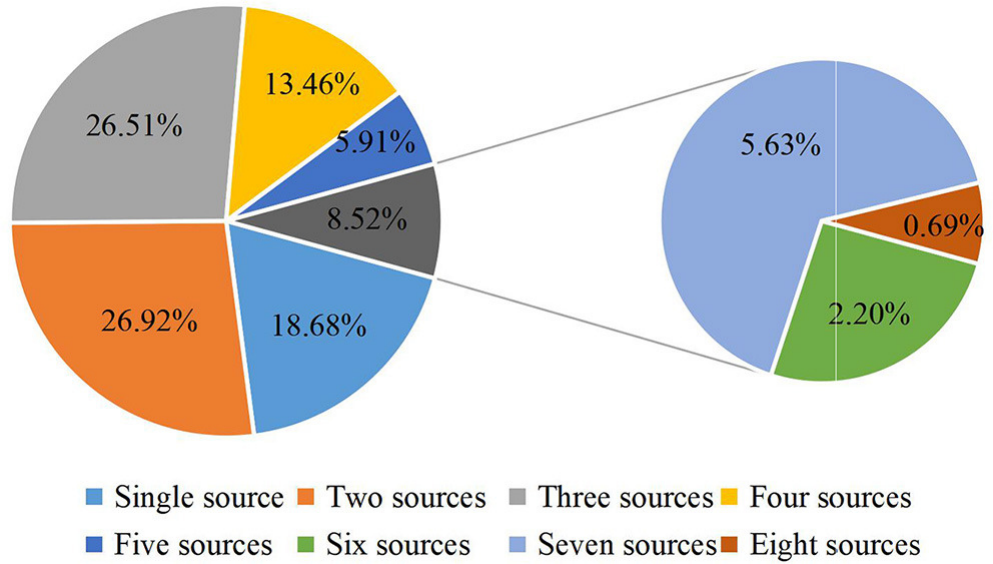
Distribution of the number of respondents' information sources.

The main source of HPV knowledge was family or friends members, school lectures and doctors, followed by the internet, subway advertising and radio/television (Figure 1). Regarding the number of information channels, respondents with two sources (26.92%) and three sources (26.51%) accounted for the majority (Figure 2).

### Multivariate Analysis

The results of the probit regression model showed that grade and doctor information source were statistically significant at the 0.05 level. The higher the grade, the lower the possibility of vaccine hesitancy. People who obtained information from the doctor had a reduced probability of vaccine hesitancy compared to other sources (Table 3). The results of marginal utility showed that the average partial effect for grade was −0.33, indicating that HPV vaccine hesitancy among master degree and above respondents was 33.00% lower than that among first grade. The average partial effect of not having a partner was −0.11, indicating that the probability of HPV vaccine hesitancy for those without a partner was 11% lower than for those with a partner, even if it is not statistically significant. The average partial effect of doctor sources of information was −0.08, indicating that respondents receiving HPV information through the Internet were 8% less likely to report vaccine hesitancy than those receiving information from other information channels.

**Table 3:** Determinants of vaccine hesitancy using Probit model.

**Hesitancy**	**Coef**.	**Std. Err**.	**z**	**P>z**	**95% CI**	**Margins**
Years of age, 16–26	−0.07	0.12	−0.53	0.60	(−0.31, 0.18)	−0.02
Male	0.12	0.11	1.06	0.29	(−0.1, 0.34)	0.04
**Grade**						
The second year of university	−0.38	0.32	−1.17	0.24	(−1.01, 0.25)	−0.10
The third year of university	−0.30	0.33	−0.92	0.36	(−0.95, 0.34)	−0.07
Fourth grade of university	−0.92	0.34	−2.73	0.01	(−1.58, −0.26)	−0.28
Fifth grade of university	−0.93	0.39	−2.42	0.02	(−1.69, −0.18)	−0.29
Master degree and above	−1.05	0.31	−3.39	0.00	(−1.66, −0.44)	−0.33
Urban	−0.11	0.12	−0.90	0.37	(−0.35, 0.13)	−0.04
No partner	−0.31	0.17	−1.85	0.07	(−0.64, 0.02)	−0.11
≤ $300	−0.04	0.11	−0.40	0.69	(−0.26, 0.17)	−0.02
Internship	0.02	0.14	0.13	0.89	(−0.26, 0.3)	0.01
**Hours of exercise (weekly)**						
1 ≤ 3 h	−0.03	0.12	−0.27	0.79	(−0.26, 0.2)	−0.01
>3 h	−0.18	0.15	−1.21	0.23	(−0.48, 0.11)	−0.06
**Information sources**						
Family or friends	0.12	0.10	1.22	0.22	(−0.07, 0.32)	0.04
Doctor	−0.23	0.11	−2.13	0.03	(−0.44, −0.02)	−0.08
Internet	−0.11	0.12	−0.95	0.34	(−0.35, 0.12)	−0.04
Radio/TV	−0.12	0.13	−0.93	0.35	(−0.36, 0.13)	−0.04
School lectures	0.02	0.11	0.21	0.83	(−0.19, 0.23)	0.01
Public welfare publicity and subway advertising	0.14	0.11	1.27	0.20	(−0.08, 0.36)	0.05
Newspapers, magazines	−0.03	0.14	−0.24	0.81	(−0.31, 0.24)	−0.01
Other	−0.35	0.23	−1.48	0.14	(−0.81, 0.11)	−0.12
_cons	1.62	0.39	4.15	0.00	(0.85, 2.38)	–

*CI, confidence interval*.

## Discussion

Our study targeted Chinese medical students and examined the incidence and reported reasons for HPV vaccine hesitancy among Chinese medical students. It also investigated the sources and number of sources of HPV vaccination information for medical students and explored the correlation between vaccination information and vaccine hesitancy.

The study found a high degree of HPV vaccine hesitancy among the people surveyed, with more than 60% of the respondents reporting hesitancy about the HPV vaccine. The findings showed that some people do not see the necessity of vaccination. This may reflect a cognitive bias, as an optimistic bias, namely, that they are unlikely to develop cervical cancer, or discounting the future, that is, lack of concern about their middle or old age in the future. Beyond this, HPV infection might be stigmatized by some as related to life misconduct. Some respondents may believe that they could avoid contracting the virus by having the same sexual partner. However, global observations suggest that 75% of sexually active adults develop HPV at some point in their lifetime (24).

Convenience was reported as an important factor in the respondents' hesitancy about the HPV vaccine. Participants reported issues with not knowing where to get reliable vaccination information (63%) and not knowing where to receive vaccination (58%). Medical students in Chongqing, China, have reported information as an important obstacle to receiving HPV vaccination (25). A total of 60% of participants reported hesitancy on the grounds of repeated vaccination doses and inconvenient vaccination. The percentage of participants who reported having to travel long distances was 60%. This may be because HPV vaccination has clear time intervals and dosage requirements. This partly increases the traffic cost of vaccination. To shorten the time cost and improve the accessibility of vaccination, vaccination sites could be added (26) by using mobile vaccination vehicles to provide vaccination services or by providing vaccination services in schools or pharmacies. Although this would improve the availability of vaccination, ensuring vaccine circulation and meeting storage standards should not be ignored; these need joint participation by the regulatory authorities. Opening online or telephone appointments is another way to make vaccination easier. SMS reminder services could also improve the rate of full vaccination.

The safety and effectiveness of vaccines was another key factor reported by medical students in vaccine hesitancy (62%). Research on the South East Asia and Western Pacific Regions has reported that safety concerns are related to vaccine hesitancy (27). Safety concerns are also related to insufficient knowledge about HPV. In fact, the safety and effectiveness of the HPV vaccine have been widely demonstrated. The Global Advisory Committee on Vaccine Safety of the WHO evaluates HPV vaccine performance as “extremely safe” (2).

The US Centers for Disease Control and Prevention noted that many people who get the HPV vaccine experience no side effects. The most common side effects are usually mild. Severe allergic reactions following vaccination are rare (28). Providing scientific information to the public is an indispensable way to reduce the impact of the vaccine controversy (29). Fear of needles (41%) is also a factor in vaccine hesitancy. Taddio's study confirmed that fear of pain is one of the major barriers to vaccination (30). In future vaccine development, the vaccine dosage type could reduce the fear of needles.

The main sources of HPV vaccine knowledge for medical students are friends/family, school lectures, doctors, and the Internet. Fewer people report newspapers, radio/television sources. A study on HPV in Italy also pointed out that fewer and fewer people get vaccination information through TV, radio or newspapers (31). Medical students who rely on family and friends for vaccine information are more likely to report vaccine hesitancy, although this result is not significant (*P* > 0.05). As parents and friends are the main source of social communication among medical students, they can always get information from them. This experience also includes views and attitudes toward the HPV vaccine. This means that, in addition to providing more vaccine information for the respondents, the popularization of the HPV vaccine information among their parents or friends may also indirectly increase respondents' knowledge of the HPV vaccine. Respondents who received information from doctors were less likely to report vaccine hesitancy (*P* < 0.05). As a professional source of information, doctors can provide authoritative HPV vaccine information to dispel people's doubts about the vaccine. A study in Florida has noted that doctors' advice on the HPV vaccine predicts vaccination status (32). Therefore, training healthcare providers to address common vaccine problems (33) may be a feasible way to reduce vaccine hesitancy. On the one hand, public health providers can provide those considering vaccinations with any information they may need (34). People who obtain information from Internet sources are less likely to report vaccine hesitancy. Young students are used to finding solutions online when experiencing problems. The Internet plays an important role in disseminating information about vaccination (35). Using the Internet as a key carrier of vaccine information may effectively increase respondents' awareness of the HPV vaccine. However, few studies also suggest that social media platforms have become a common source of vaccine information and false information (36). To reduce the spread of false information, it is necessary to increase the publication of accurate vaccine information and the supervision of online information.

HPV vaccine hesitancy needs to be given more attention, especially among medical students and medical staff. These people are not only HPV vaccine recipients, but also a key force in promoting HPV vaccine coverage. The widespread occurrence of HPV vaccine hesitancy will affect vulnerable populations including newborns and pregnant women. This study shows that the uncertainty of the safety of HPV vaccines is the reason why medical students hesitate to produce vaccines. Concerns about vaccine safety have also appeared in the hesitancy of COVID-19 vaccine studies in the United States and Canada (37, 38).

It is necessary to carry out health education activities on the safety and effectiveness of vaccines. This may be an effective way to reduce the misunderstanding of HPV vaccines by medical students and the public and promote uptake of HPV vaccines. The target population of health education can be parents, teachers, public health service providers, specialist doctors and vaccinators themselves, and these people can also become implementers of health education. Strategies are as follows: (1) Providing HPV vaccine information. When the vaccination audience is vaccinated with other vaccines, vaccination service providers take the initiative to provide information about HPV vaccines (39). Distributing vaccine brochures at vaccination sites, posting vaccine posters, providing vaccine appointment explanation services, etc.; (2) Health care providers conduct health lectures in schools to explain the knowledge and preventive measures of cervical cancer and genital diseases; (3) Doctors can also promote correct information to the right-age vaccinated population and their parents, and encourage this population to be vaccinated. (4) Health professionals or young doctors have often been interviewed to get quick and easy guidance on procedures or processes that could be transferred to the general population (40) or to quickly recruit subjects on which verify individual's personal experiences and sociocultural factors (41). In addition to increasing awareness of HPV vaccines through health education, communication on the risks of HPV vaccines can help build public confidence and trust. The vaccine management party or the health department can set up a risk communication department to conduct preventive risk communication. Avoid increasing vaccine hesitancy due to asymmetric risk information.

Limitations of this study include that there was no segmentation of vaccine hesitancy measurements and that we gathered information only on whether vaccine hesitancy occurred but not on the frequency and degree of vaccine hesitancy. The measurement of the degree is a very complicated issue. Even if there are HPV vaccine hesitancy scales developed in other countries, the applicability of the scales in China has yet to be verified. Using Likert scale and other tools to measure the degree of hesitancy will be our next research content. The study included 728 participants, and the study may have representative questions. However, the number of medical students in China is limited, and we have distributed the samples to different regions of China as much as possible. Another limitation was the cross-sectional design, which cannot provide causal relationships. Despite these limitations, the survey gained a high response rate, showing a strong interest in the HPV topic.

## Conclusions

Vaccine hesitancy is prevalent among Chinese medical students. Medical students are potential beneficiaries of the HPV vaccine and a key force for future vaccination; thus, it is necessary to implement measures that address the reasons for their reported vaccine hesitancy. Making vaccination convenient by the use of mobile vaccination vehicles and increasing vaccination sites such as in schools or pharmacies could be an effective way to reduce HPV vaccine hesitancy. The role of medical workers in expanding respondents' understanding of the safety and effectiveness of the vaccine may also help reduce vaccine hesitancy. Additionally, the role of online media in vaccine hesitancy should also be fully considered.

## Data Availability Statement

The raw data supporting the conclusions of this article will be made available by the authors, without undue reservation.

## Ethics Statement

The study was conducted according to the guidelines of the Declaration of Helsinki, and approved by the Harbin Medical University School of Health Management and Institutional Research Board (reference no. HMUIRB20210006, approved on 30 June 2021). Written informed consent for participation was not required for this study in accordance with the national legislation and the institutional requirements.

## Author Contributions

LZ, JW, YL, PC, XZ, and GL: conceptualization. LZ, JW, YL, and PC: methodology, data curation, and formal analysis. LZ, XZ, and GL: validation. YL, PC, JW, and XZ: investigation. XZ and GL: resources, supervision, and project administration. LZ: writing—original draft preparation. XZ: writing—review and editing. YL, PC, and JW: visualization. All authors contributed to the article and approved the submitted version.

## Conflict of Interest

The authors declare that the research was conducted in the absence of any commercial or financial relationships that could be construed as a potential conflict of interest.

## Publisher's Note

All claims expressed in this article are solely those of the authors and do not necessarily represent those of their affiliated organizations, or those of the publisher, the editors and the reviewers. Any product that may be evaluated in this article, or claim that may be made by its manufacturer, is not guaranteed or endorsed by the publisher.
