# Supplementary material for: HPV Vaccine Hesitancy Among Medical Students in China: A Multicenter Survey
Source: Front Public Health. 2022 Feb 21;10:774767. doi: 10.3389/fpubh.2022.774767 (PMC8900914; doi:10.3389/fpubh.2022.774767)
Supplement: Supplementary file 1 [file Data_Sheet_1.DOCX]

**Additional file 1.**

Investigation on Hesitancy and Willingness to Pay for Human Papillomavirus (HPV) Vaccine

Dear classmates:

The HPV vaccine is the first human attempt to eradicate a kind of cancer through a vaccine, which is of epoch-making significance. In order to better understand the hesitancy and willingness to pay for HPV vaccines among college students, we conducted this study. It takes about 5-8 minutes to fill out this questionnaire. The results of the survey are only used for scientific research and will not have any impact on individuals. Filling in each questionnaire will contribute to the elimination of cancer. Please be sure to fill in the questionnaire truthfully. If you encounter any problems during the filling process, you can contact 188468***** for consultation. (***Please fill out the questionnaire completely voluntarily. If you cannot complete the questionnaire for some reason, you can exit the questionnaire at any time.**)

**Ⅰ. Basic situation investigation**

1. Your gender: [Single choice] *

○male ○female

2. Your age: [Single choice ] *

○15 years old and below

○16-26 years old

○27-45 years old

○46 years old and above

3. Your major [Fill in the blanks] *

Such as: clinical medicine

_________________________________

4. Your schoo [Fill in the blanks] *

_________________________________

5. Your grade [Single choice] *

○ Freshman

○ Sophomore

○ Junior

○ Senior

○ University Grade 5

○ Graduate student and above

6. Current residential address: [Fill in the blanks] *

_______________________________

7. Type of your family residence: [Single choice] *

○City ○Rural

8. Your nationality: [Single choice] *

○Han ○Other

9. Marital status: [Single choice] *

○Unmarried

○Married

○Divorce

○Widowed

○Other

10. Have you ever gone to hospital, disease control, or other departments of the health system for internships/trainees: [Single choice] *

○Yes

○No

11. How much is your monthly daily consumption of ______ : [Single choice] *

○Below 1000 yuan

○1000-1999 yuan

○2000-2999 yuan

○3000-3999 yuan

○4000 yuan and above

12. The time when you browse the Internet for news and news every day: [Single choice] *

○≤1 hour

○1-3 hours

○≥3 hours

13. How long do you exercise each week ______ hours: [Single choice] *

○≤1 hour

○1-2 hours

○2-3 hours

○≥3 hours

14. Is there a fitness plan recently: [Single choice] *

○Yes

○No

**Ⅱ.Investigation of vaccine hesitancy**

Vaccine hesitancy, that is, delaying or postponing vaccination when it is available, risks reversing the progress made in tackling vaccine-preventable diseases. Assuming that the HPV vaccine is available, you are asked to answer the following questions with your honest answers.

15. Have you ever been hesitant to produce the HPV vaccine: [Single choice] *

○Yes

○No (Please skip to question 56)

1. Reasons for vaccine hesitancy [Matrix Single Choice Questions] *

| Items | Yes | No |
| --- | --- | --- |
| No vaccination was considered necessary |  |  |
| Don't know where to get vaccinated |  |  |
| The vaccination site is far away and requires a long journey |  |  |
| Need fractional inoculation, feel troublesome |  |  |
| Don't know where to get good/reliable information |  |  |
| Hear or read negative news |  |  |
| Fear of needles |  |  |
| Someone told me they had a bad reaction |  |  |
| Had a bad experience with previous vaccinations due to health clinics/vaccinators |  |  |
| Worried about vaccine safety/side effects |  |  |
| I've been told that vaccines aren't safe |  |  |
| Vaccination had bad physiological and psychological reactions before |  |  |
| Health care workers' attitudes towards the HPV vaccine |  |  |
| Religious reasons/other beliefs |  |  |

17. Do you have any other reason other than the above options: [Fill in the blanks] *

_________________________________

18. Please list other reasons why others have delayed or refused HPV vaccination. [Fill in the blanks] *

___________________________________________________________

19. Where did you get the relevant knowledge of HPV virus and its vaccine: [Multiple Choice ] *

□Doctor consultation

□Introduced by family or friends

□Hospital/School Public Lectures

□Social welfare promotion (subway advertising, etc.)

□Radio/TV

□Newspaper/Magazine

□Network

□Other _________________
