# Supplementary material for: HPV Vaccine Hesitancy Among Medical Students in China: A Multicenter Survey
Source: Front Public Health. 2022 Feb 21;10:774767. doi: 10.3389/fpubh.2022.774767 (PMC8900914; doi:10.3389/fpubh.2022.774767)
Supplement: Supplementary file 2 [file Data_Sheet_2.DOCX]

**Additional file 2.**

**Confidence, Complacency, Convenience Model of Vaccine Hesitancy**

- In the “3Cs” model, **confidence** is defined as trust in 1) the effectiveness and safety of vaccines; 2) the system that delivers them, including the reliability and competence of the health services and health professionals and 3) the motivations of the policy-makers who decide on the needed vaccines.
- Vaccine **complacency** exists where perceived risks of vaccine-preventable diseases are low and vaccination is not deemed a necessary preventive action. Complacency about a particular vaccine or about vaccination in general is influenced by many factors, including other life/health responsibilities that may be seen to be more important at that point in time. Immunization program success may, paradoxically, result in complacency and ultimately, hesitancy, as individuals weigh risks of vaccines against risks of diseases that are no longer common. Self-efficacy (the self-perceived or real ability of an individual to take action to vaccinate) also influences the degree to which complacency determines hesitancy.
- Vaccine **convenience** is measured by the extent to which physical availability, affordability and willingness-to-pay, geographical accessibility, ability to understand (language and health literacy) and appeal of immunization services affect uptake. The quality of the service (real and/or perceived) and the degree to which vaccination services are delivered at a time and place and in a cultural context that is convenient and comfortable also affects the decision to be vaccinated and could lead to vaccine hesitancy.

Reference

World Health Organization . Report of the Sage Working Group on Vaccine Hesitancy. (2014).
